# ﻿*Cyphocarpusperennis* (Asterales, Campanulaceae, Cyphocarpoideae), a new species endemic to the Andes of the Atacama Desert, Chile

**DOI:** 10.3897/phytokeys.259.155414

**Published:** 2025-06-27

**Authors:** Ludovica Santilli, Natali Cruz, Claire De Schrevel, Philippe Dandois, Nicolás Lavandero, Maria Fernanda Perez

**Affiliations:** 1 Santiago, Chile Unaffiliated Santiago Chile; 2 Comunidad Indígena Colla Dos Álamos, Tierra Amarilla, Copiapó, Chile Comunidad Indígena Colla Dos Álamos Copiapó Chile; 3 Copiapó, Chile Unaffiliated Copiapó Chile; 4 Facultad de Ciencias Biológicas, Pontificia Universidad Católica de Chile, Avenida Libertador B. O’Higgins 340, Santiago, Chile Pontificia Universidad Católica de Chile Santiago Chile

**Keywords:** Andes, biodiversity, Cyphocarpoideae, phylogeny, taxonomy

## Abstract

A new species, *Cyphocarpusperennis***sp. nov.**, endemic to the Andes of the Atacama Desert of Chile is here described. Based on new nrDNA ITS sequence data, phylogenetic analyses place this novel species as sister to the remaining species of the genus. *Cyphocarpusperennis* presents unique characters within the genus, such as a perennial habit and a compact leafy rosette. A detailed description, distribution map, insights about its habitat, conservation status, photographs and an updated key to *Cyphocarpus* are provided. As for most species found in the Atacama Region, mining activities and climate change represent a major threat for its conservation.

## Abstract (es)

Se describe una nueva especie, *Cyphocarpusperennis***sp. nov.**, endémica de los Andes del Desierto de Atacama de Chile. Con base en nuevos datos de secuencias de rnADN ITS, análisis filogenéticos sitúan la nueva especie como hermana del resto de las especies del género. *Cyphocarpusperennis* posee caracteres únicos dentro del género, tales como un hábito perenne y hojas organizadas en una roseta compacta. Se provee una descripción morfológica detallada, un mapa de distribución, información sobre el hábitat, estado de conservación, fotografías y una clave dicotómica actualizada para la identificación de las especies de *Cyphocarpus*. Como es el caso de varias especies altoandinas de la región de Atacama, las actividades mineras y el cambio climático representan las mayores amenazas a su conservación.

## ﻿Introduction

*Cyphocarpus* Miers is a genus composed of three currently described species within the family Campanulaceae which are all endemic to northern Chile. The genus was first described by Miers (1848) based on specimens collected by Thomas Bridges in the region of Coquimbo, near Paihuano (30°01'S 70°31'W). Miers recognized the uniqueness of its morphology and described the subfamily Cyphocarpoideae, placing the first described species, *Cyphocarpusrigescens* Miers, within the class Campanulae (sensu Jussieu 1789), based on the epigynous corolla and alternipetalous stamens. Sandwith (1931) described the second species of the genus, *Cyphocarpusinnocuus* Sandwith, based on specimens collected by Elliot and Gourlay in 1927 in Andacollo valley, Coquimbo region (30°13’ S 71°04’ W), at approximately 1000 m above sea level (a.s.l.). The last species, *Cyphocarpuspsammophilus* Ricardi, was described in 1959 based on specimens collected the same year in the locality of Cachiyuyo, Huasco, Atacama region (29°02'S, 70°53'W) at approx. 800 m a.s.l.

The most comprehensive systematic work made of *Cyphocarpus* was done by Hansen (2016), who tested the historical hypotheses concerning the evolution of Campanulaceae and the placement of Cyphocarpoideae Miers using chloroplast genomes and the nuclear ribosomal cistron. The study included sampling of all five subfamilies and all three extant species of *Cyphocarpus*. According to Hansen (2016), the monogeneric subfamily Cyphocarpoideae is sister to Nemacladoideae, which, together with Lobelioideae, forms an early divergent lineage within Campanulaceae. The author suggests that the distribution of Cyphocarpoideae can be explained by a dispersal from either the Nearctic or Neotropics (Hansen 2016).

Regarding its morphology, alike all Campanulaceae, *Cyphocarpus* possesses latex-producing laticifers both in vegetative and floral organs (Lammers 1992). It also has zygomorphic, bilabiate corollas, similar to Lobelioideae, Cyphioideae, and some Nemacladoideae. However, unlike the aforementioned clades, in Cyphocarpoideae, the upper lip is composed of a single corolla lobe that forms a hood whose margins are connivent so that it remains tightly closed. The margin is winged at the distal end of the hood, the wings fuse and form an appendage that projects off the hood (Hansen 2016). Another distinctive character of the genus is the presence of three prominent yellow ridges at the palate which are the result of induplicate estivation of the corolla lobes.

According to the protologues, field and herbarium observations, all *Cyphocarpus* species are annual herbs and to date they are considered to be endemic to Chile. This is not surprising as Chile is known to harbour one of the 35 world biodiversity hotspots, given its high number of endemic species and high levels of threat (Myers et al. 2000; Mittermeier et al. 2004). This hotspot called “Chilean winter rainfall-Valdivian forests”, is mostly located in central Chile with its northern distribution placed in the Atacama region (Arroyo et al. 2004). The climate in the region is marked by low precipitation during the winter, low relative humidity inland, and strong daily variation in temperature.

The issues with conservation are particularly critical for central Chile and the “Norte Chico”, with less than 5% of the territory under protection and the pressure of human activity such as overgrazing, burning, land clearance for agriculture and mining activities (Squeo et al. 2001; Arroyo et al. 2006; Armesto et al. 2007). Moreover, climate change predictions estimate an increase of up to 5 °C in temperature in the high elevations of the Andes and a decrease in precipitations by 2065 (Juliá et al. 2008) which may affect the vegetation of these elevations.

Thus, it is of fundamental importance to systematically describe new species unknown to science, as conservation efforts cannot be effective without an exhaustive inventory and a deep understanding of the biodiversity that composes the ecosystems we aim to protect.

The aim of the present study is to describe a new species of *Cyphocarpus* from the Andes of the Atacama Desert, in northern Chile, study its phylogenetic position, describe its distribution and habitat, providing a preliminary conservation assessment according to the IUCN criteria, as well as a dichotomous key of the genus.

## ﻿Methods

### ﻿Herbarium studies and fieldwork

During the austral summer of 2024, several botanical expeditions were made near to the locality of Morros Negros, Tierra Amarilla district, Atacama Region, Chile. Specimens that could not be assigned to any known species were found. Recordings of observations of the habitat were made in the field along with collection of herbarium material, pickled specimens in ethanol 70%, preservation of leaf tissues for molecular analyses in silica gel and measurements of plant size. All collections were obtained with the permission of the Comunidad Indígena Dos Álamos, the Colla indigenous community who owns the land where the putative new species was collected. All specimens collected during fieldwork were deposited in SGO and CONC (acronyms following Thiers 2025). Besides the revision of the herbaria material (CONC, ULS, SGO), a thorough search was performed on iNaturalist (https://www.inaturalist.org/) in order to find possible observations that matched the morphology of the new species.

### ﻿Localities

**Figure 1.**
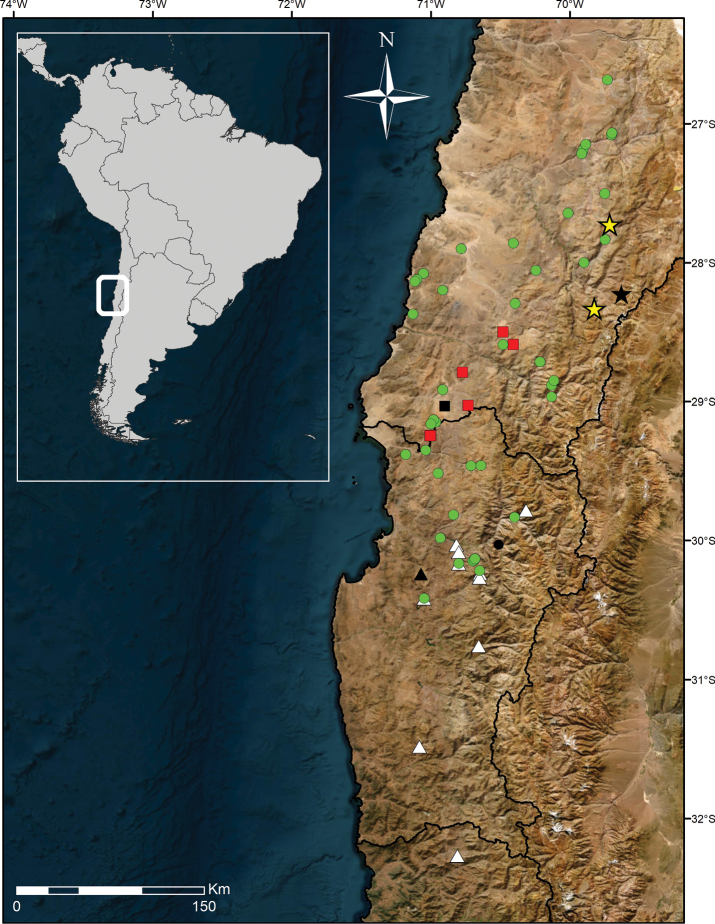
Distribution map of *Cyphocarpus*. Green circles: *C.rigescens*; white triangles: *C.innocuus*; red squares: *C.psammophilus*; yellow stars: *C.perennis.* Type localities for each species are shown in black. Black line indicates boundaries between administrative regions of Chile and the borderline between Chile and Argentina. Service Layer Credits: Esri Maxar Earthstar Geographics and the GIS user community.

The locality of collection of the type specimen is situated at approximately 19 km southeast of Juntas del Potro, in the district of Tierra Amarilla, located in the central east of the Atacama region, nearly 100 km from Copiapó and 120 km from Vallenar at ca. 3637 m a.s.l. (Fig. 1). To characterise the area, we consulted the bioclimatic and vegetation classification of Chile by Luebert and Pliscoff (2017). According to this classification, the locality corresponds to a low tropical-mediterranean Andean shrub dominated by *Adesmiahystrix* Phil. and *Ephedrabreana* Phil. Additionally, we reviewed iNaturalist observations, which, although not based on collected specimens, provide georeferenced data useful for expanding knowledge of the species’ distribution. The localities from iNaturalist, whose specimens have not been collected yet, are both found in the district of Tierra Amarilla, specifically at approximately 24 km south of Iglesia Colorada, along route C-463 (https://www.inaturalist.org/observations/50569276), and approximately 17 km south of Carrizalillo along route C-503 (https://www.inaturalist.org/observations/50569279). Both localities belong to the low tropical-desertic Andean shrub dominated by *Atripleximbricata* D.Dietr. The Atacama Region is found between latitudes 26°–29° S, the climate is characterized by low precipitation concentrated in a limited period of time during the winter season, a strong decrease in relative humidity inland, and a considerable daily variation in the atmospheric temperature (Juliá et al. 2008).

### ﻿Morphological analysis

Morphological analyses were based on both fresh and dried specimens collected by the authors during field trips in December 2024. Photographs of living plants were taken in the field. To accurately describe the species, plants were dissected and observed under a stereoscope. Terminology for describing plant morphology follows Beentje (2016) and Hansen (2016). All measurements were performed using an analog caliper, and both minimum and maximum values for each character were recorded to capture the full range of variation. The selected traits were chosen based on their relevance to species identification and differentiation within the genus *Cyphocarpus*. These characters are commonly used in taxonomic studies to distinguish species and are consistent with previous morphological analyses in related taxa.

### ﻿Conservation status

The preliminary assessment of the conservation status of the species was made using the International Union for Conservation of Nature (IUCN 2012) categories and criteria, following the most recent guidelines (IUCN Standards and Petitions Committee 2024). The extent of occurrence (EOO) and area of occupancy (AOO) were calculated using GeoCat (Bachman et al. 2011).

### ﻿DNA extraction, amplification, sequencing, and phylogenetic analyses

Total genomic DNA from the holotype of the new species (*CDS-931*, SGO) was extracted from silica-dried material using the Qiagen DNeasy Plant Mini Kit (QIAGEN) following the manufacturer’s instructions. Genomic DNA was used to amplify by PCR a partial region of the nuclear ribosomal DNA (nrDNA), comprising a region of the 18S rRNA gene, the internal transcribed spacer 1 (ITS1), the 5.8S rRNA gene, the internal transcribed spacer 2 (ITS2) and a partial region of the 26S rRNA gene. Amplification was performed using the primers ITS4 and ITS5 (White et al. 1990). The PCR thermocycling conditions consisted of 95° C for 5 min of initial denaturation, followed by 30 cycles of 95° C for 1 min, 55° C for 1 min, and 72° C for 1.5 min, and a final extension step of 72° C for 15 min. We amplified this region in 25 μL PCR reactions using 12.5 μL GoTaq® G2 Green Master Mix (PROMEGA), 1 μL of each primer (10 μM), 1.25 μL BSA, 6.75 μL nuclease-free water, and 2.5 μL of DNA (~100 ng/μL). Sanger sequencing was performed using the same primers for amplification. Sequencing was performed in the Plataforma de Secuenciación y Tecnologías Ómicas, Pontificia Universidad Católica de Chile, using the ABI PRISM 3500 xl Genetic Analyzer (Applied Biosystems™). Forward and reverse sequences were assembled using Geneious Prime 2022.2.1 (https://www.geneious.com). As a preliminary analysis, we BLASTed (https://blast.ncbi.nlm.nih.gov/) the assembled sequence, obtaining the highest match to *Cyphocarpusrigescens* (90.85%). Having confirmed its affinity to *Cyphocarpus*, we extracted and amplified with the aforementioned methods one specimen from the three other species of the genus (*C.rigescens*, *C.innocuus* and *C.psammophilus*). GenBank accession numbers for all newly generated DNA sequences and voucher information are given in Suppl. material 1.

Phylogenetic analyses included a sampling of the closest genera of *Cyphocarpus*, according to Hansen (2016). These include *Nemacladus* and *Pseudonemacladus*, from the Nemacladoideae subfamily. As an outgroup, we used *Codonopsispilosula* (Campanuloideae). A broader sampling within Campanulaceae could not be performed due to the high degree of divergence of the ITS sequences, which complicated the assessment of homology in the alignment. The assembled sequences generated by this study and the sequences downloaded from Genbank (https://www.ncbi.nlm.nih.gov/genbank/) were aligned using the MAFFT v.7.490 (Katoh et al. 2002; Katoh and Standley 2013) algorithm in Geneious Prime 2022.2.1 (https://www.geneious.com). Phylogenetic analyses were performed for both Maximum-likelihood (Felsenstein 1981), using RAxML-SSE3 version (Stamatakis 2014) included in RAxMLGUI v.2.0.10 (Edler et al. 2021) and Bayesian inference using MrBayes x64 v3.2.7 (Ronquist et al. 2012), respectively. The gaps and indels were treated as missing data. The GTR+I+G model of nucleotide sequence evolution was determined based on the Akaike information criterion (AIC) given by MrModeltest v2 (Nylander 2004). Maximum likelihood analyses were run using the GTRGAMMA approximation, which approximates to a GTR model. The analysis included 1000 ML slow bootstrap replicates with 500 runs. Bayesian analyses were conducted under the GTR+I+G model, with two independent runs for 1 million generations, sampling every 1000 generations. Time series plots and effective sample size (ESS) were analysed using TRACER v.1.7 (Rambaut et al. 2018) to check convergence for each run. The first 250.000 generations were discarded as burn-in.

## ﻿Results

**Figure 2.**
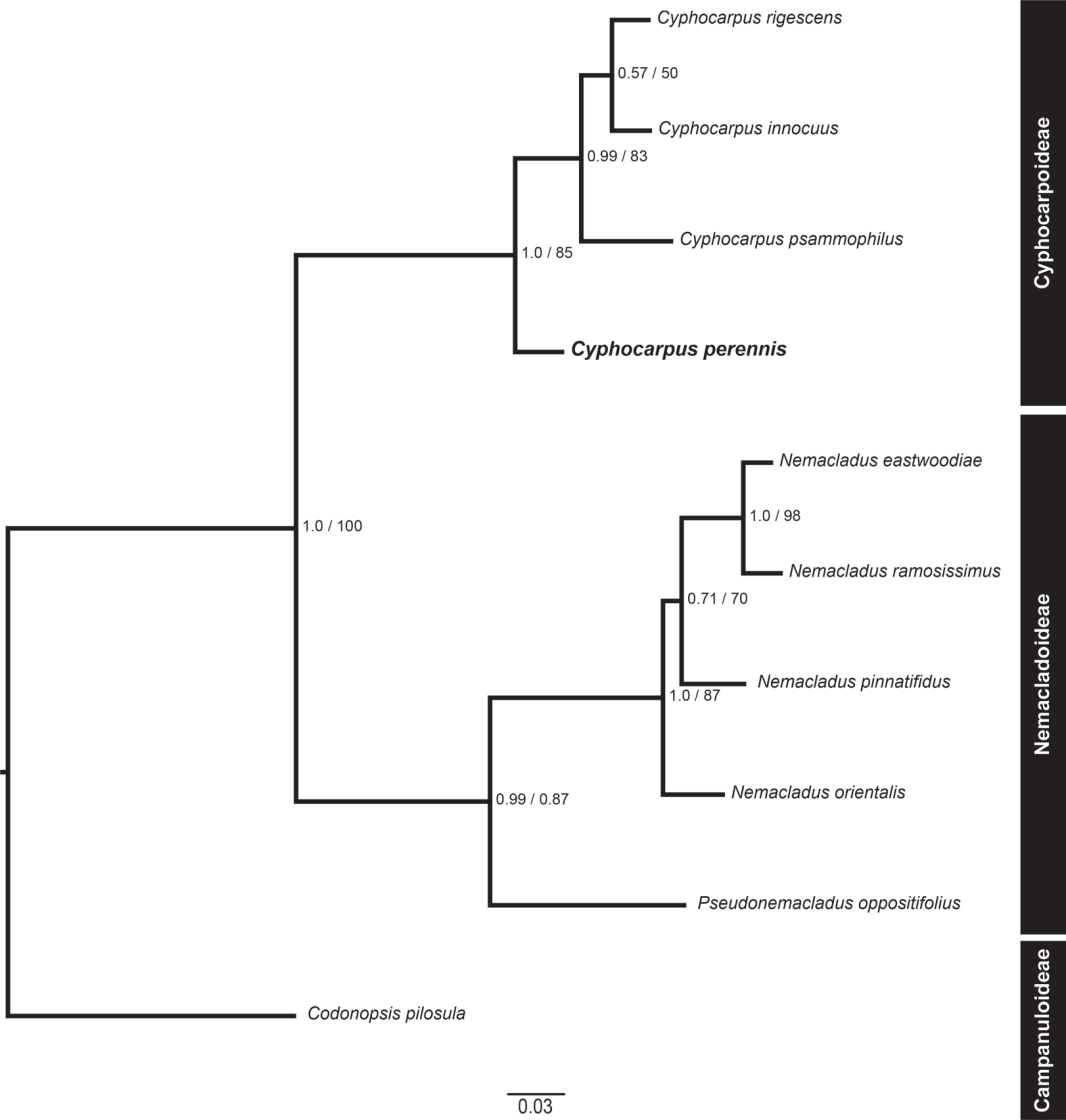
Phylogeny of *Cyphocarpus* resulting from Bayesian analysis of the ITS region. Branch lengths indicate substitutions/site. Numbers next to the nodes represent the posterior probabilities from the Bayesian analysis and bootstrap values from the Maximum Likelihood analysis (PP/BS). The new species *Cyphocarpusperennis* is shown in bold.

The ITS nucleotide matrix, representing a total of 4 ingroup and 6 outgroup accessions, contained 701 characters. The alignment contained 416 (59.3%) identical sites and 115 parsimony-informative sites. The topology of both Bayesian and Maximum likelihood analyses yielded congruent topologies (Fig. 2), recovering a strongly supported monophyletic Cyphocarpoideae (PP = 1, BS = 85) as sister to monophyletic Nemacladoideae (PP = 0.99, BS = 0.87), also with strong support. Within Cyphocarpoideae, the new species, *Cyphocarpusperennis*, appears as sister to the rest of *Cyphocarpus* (PP = 0.99, BS = 83). *Cyphocarpuspsammophilus* appears as sister to the clade formed by *Cyphocarpusinnocuus* and *Cyphocarpusrigescens*, although the latter clade has low support (PP = 0.57, BS = 50). These results confirm the phylogenetic affinity of this new species to the genus *Cyphocarpus*.

### ﻿Taxonomic treatment

#### ﻿ Cyphocarpus perennis

Taxon classificationPlantaeAsteralesCampanulaceae

Santilli & Lavandero sp. nov.

85E8E8DF-CF70-56D1-9252-67BA1D7157D2

urn:lsid:ipni.org:names:77364408-1

Figs 3 , 4 , 5

##### Type.

Chile • Atacama: Copiapó, Tierra Amarilla, Cerro Morros Negros, 28°13'42"S, 69°37'48"W, 3,613 m, 27 December 2024, fl., *Claire de Schrevel, Natali Cruz & Philippe Dandois CDS-931*, (holotype SGO 171975!; isotype CONC).

##### Diagnosis.

*Cyphocarpusperennis* differs to other *Cyphocarpus* species due to its perennial habit, rosette-like growth form, rounded leaves, and tomentose indumentum.

##### *Description*.

Perennial herb, forming groups of seasonally persistent annual rosettes, up to 3 cm tall and 4 cm wide. ***Rhizome*** creeping to extended, lignified and branching, buried several centimetres below the substrate. ***Stems*** emerging from the nodes of the rhizome, almost entirely subterraneous, belowground portion up to 15 cm long, glabrous, bearing leaves reduced to scales, aerial portion of the stem pubescent (same indumentum of the leaves), up to 3 cm long. ***Leaves*** flabellate, densely strigose to hirsute, trichomes subulate, petioles up to 5 times as long as the lamina, approx. 15 mm long, arranged in short internodes. ***Lamina*** as long as wide, up to 6 mm long and 6 mm wide, crisped; base attenuate towards the petiole; apex rounded; margin crisped, bidentate. ***Inflorescence*** an axillary solitary flower, subtended by small leaflike bracts. ***Flowers*** pentamerous, sessile, epigynous, zygomorphic, bilabiate, hermaphrodite, protandrous. ***Sepals*** 5, free, linear, ⅕ of corolla tube length, same indumentum as leaves, margin entire. ***Petals*** 5, hirsute outside, glabrous inside, basally fused into a 13–15 mm long corolla tube, then free; lobes white, the upper lip comprised of a single corolla lobe, ca. 4 mm long, that forms a hood with connivent, winged margins; wings fused at the distal end of the hood to form a small appendage that slightly projects off the hood; the remaining 4 ventral corolla lobes, ca. 5 mm long, forming a palate ca. 11 mm wide, with three prominent yellow ridges at the base, alternating with the lobes. ***Stamens*** 5, epipetalous, haplostemonous, free portion of the filaments ca. 3 mm, pubescent, anthers included in corolla tube, connivent around the style, longitudinally dehiscent. ***Ovary*** inferior, hirsute, cylindrical, syncarpous; carpels 2, locules 1, placentation parietal, style 1, 16–17 mm, stigma globose, 1 × 2 mm, slightly two-lobed, included in the hood and eventually protruding from it. ***Fruit*** a rounded capsule, 10–11 × 10 mm, hirsute, crowned by persistent sepals, becoming papery and releasing the seeds by irregular longitudinal ruptures. ***Seeds*** ca. 2 mm long, cylindrical and slightly curved, pale yellow to brown, surface ribbed longitudinally.

**Figure 3.**
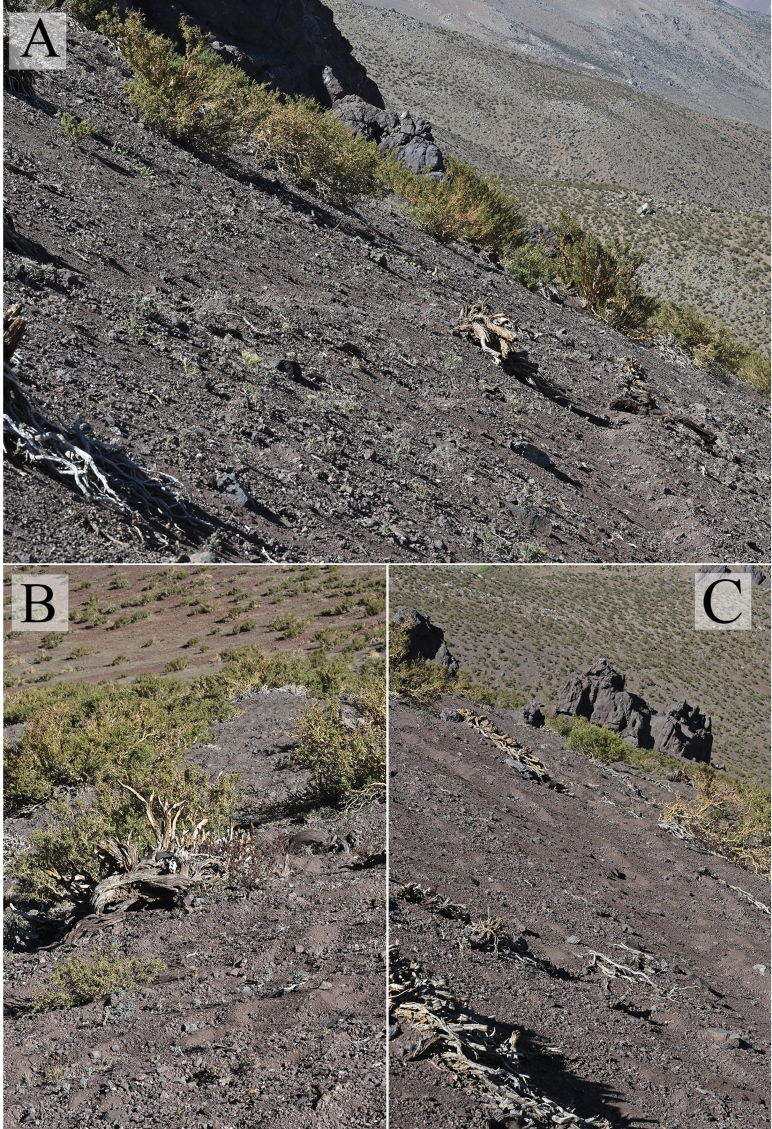
*Cyphocarpusperennis*. **A–C.** Habitat of *Cyphocarpusperennis* showing gravelly slopes dominated by *Adesmiahystrix*.

##### Etymology.

The specific epithet refers to the perennial habit which distinguishes the novel species from the other species of the genus, which are all annual herbs.

##### Phenology.

Flowering from late November to early January.

##### Distribution and habitat.

Endemic to the eastern Andean ranges of the Atacama Desert, in the municipality of Tierra Amarilla. It is known from the localities of Morros Negros, Carrizalillo and Iglesia Colorada (Fig. 1). The species is recorded to grow at high elevations between approx. 2900 and 3700 m, in loose gravelly and sandy soil, at full sun exposure.

**Figure 4.**
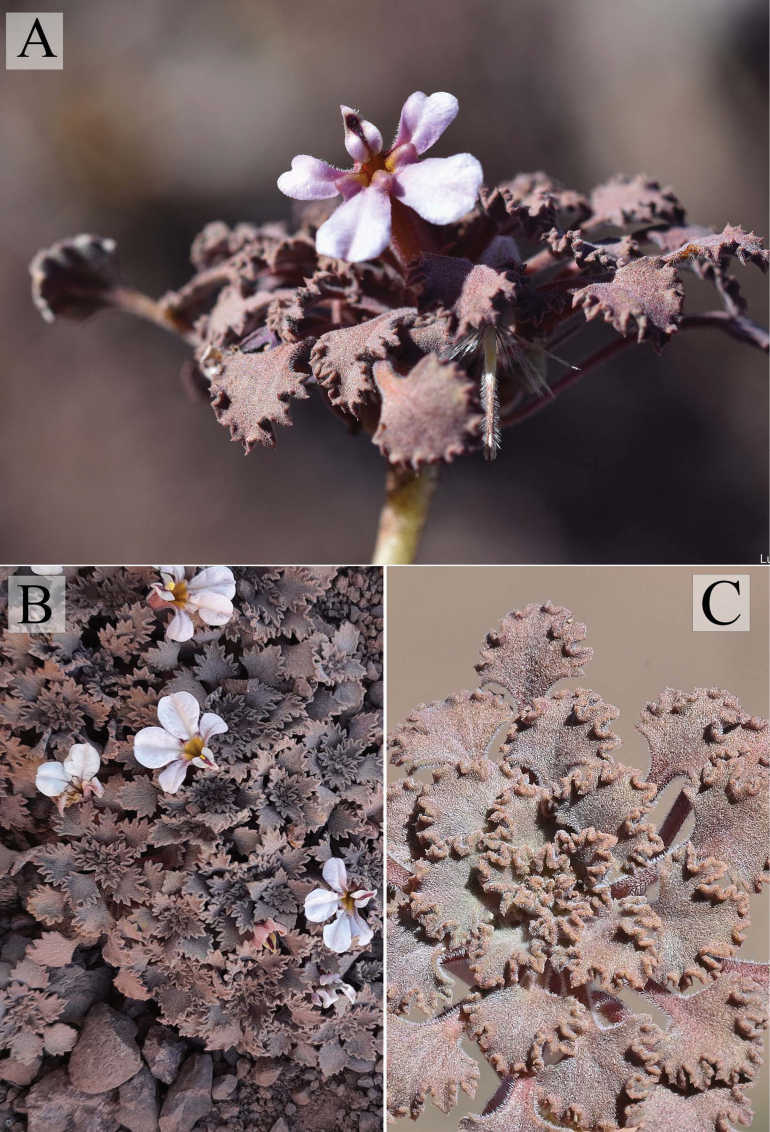
*Cyphocarpusperennis.***A.** General view of the plant; **B.** Upper view of the plant; **C.** Details of the vegetative rosette.

##### Associated vegetation.

*Cyphocarpusperennis* has been observed at elevations near the upper limit of the arborescent growth, represented by a scrub of *Adesmiahystrix* Phil. Accompanying species recorded in the field include *Adesmiahystrix*, *Menonvilleacuneata* (Gillies & Hook.) Rollins, *Doniophytonweddellii* Katinas & Stuessy, *Pachylaenaatriplicifolia* D. Don ex Hook. & Arn.

**Figure 5.**
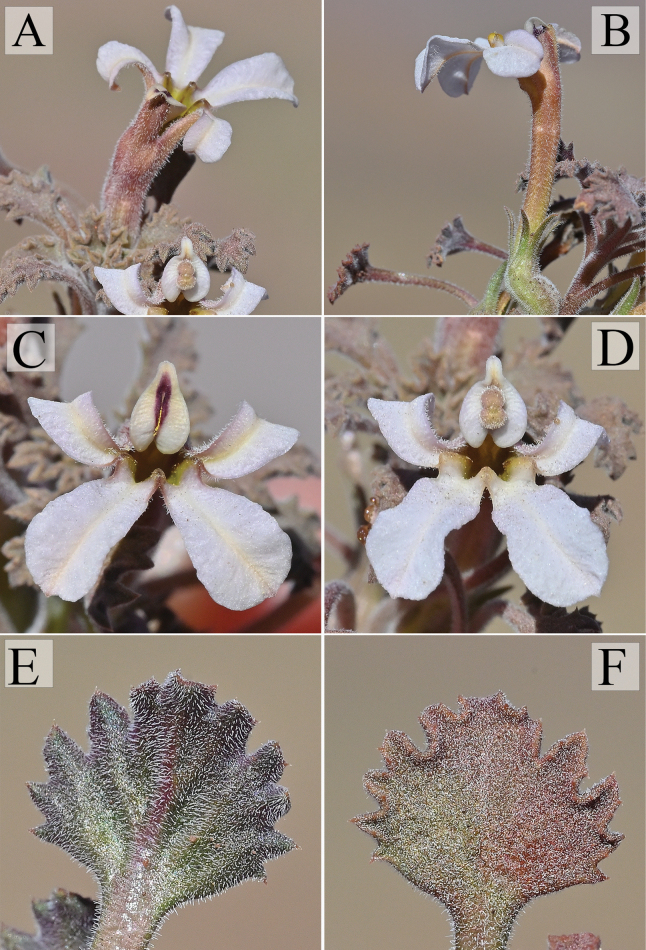
*Cyphocarpusperennis.***A.** Upper view of a flower; **B.** Lateral view of a flower showing corolla tube and calyx; **C.** Frontal view of a flower during the male phase of the protandry; **D.** Frontal view of a flower during the female phase of the protandry; **E.** abaxial side of a leaf; **F.** adaxial side of a leaf.

##### Conservation status.

*Cyphocarpusperennis* is assessed here as Endangered (EN) under the IUCN categories and criteria B1ab(iii) + 2ab(iii). Criteria B1 and B2 were selected because its extent of occurrence (EOO) is < 5,000 km^2^ (588.908 km^2^) and its area of occupancy (AOO) is < 500 km^2^ (12 km^2^). Criterion “a” was selected because it is known to exist in only three locations, criterion “b(iii)” was selected because there is a projected decline in the area, extent, and quality of habitat. According to Luebert and Pliscoff (2017), the low tropical-desertic Andean scrub is not represented in the national system of protected area and its extent is projected to decrease by 48.45% under the IPCC AR5 scenario 2.6, and by 63.37% under the scenario 8.5 for the period 2040–2070. Similarly, the low tropical-mediterranean Andean scrub dominated by *Adesmiahystrix* and *Ephedrabreana* is expected to decrease its extent by 27.21% under the IPCC AR5 scenario 2.6 and by 30.05% under the scenario 8.5 for the period 2040–2070. Additionally, high Andean plants are particularly sensitive to global warming and the reduction in snow cover and precipitation is worsened by the increase of temperatures which are predicted to keep rising by models of climate change (Juliá et al. 2008; Cordero et al. 2019). Moreover, *Cyphocarpusperennis* is found in the second region with more mining concessions in Chile (after Antofagasta), with more than 8,918 concessions and nearly 2,500,000 ha under this real estate right (SERNAGEOMIN 2024). All localities where the species is found are under mining concessions (https://catastro.sernageomin.cl/). Finally, *Cyphocarpusperennis* is not present in any statal or private protected area in Chile. It is important to highlight that the type locality of the species is within the boundaries of the indigenous Colla community of Dos Álamos, and permission must be requested to enter.

##### Additional material examined.

**Chile** • **Atacama Region: [Copiapó Province**] Cerro Morros Negros 28°13'37"S, 69°37'45"W, 3637 m, 27 Dec 2024, *Claire de Schrevel, Natali Cruz & Philippe Dandois CDS-929* (SGO!); • Cerro Morros Negros 28°13'38"S, 69°37'45"W, 3636 m, 27 Dec 2024, *Claire de Schrevel, Natali Cruz & Philippe Dandois CDS-930* (SGO!); • Cerro Morros Negros 28°13'42"S, 69°37'48"W, 3613 m, 27 Dec 2024, *Claire de Schrevel, Natali Cruz & Philippe Dandois CDS-932* (SGO!); • 24 km south of Iglesia Colorada, along route C-463, 27°43'22"S, 69°42'47"W, 9 Jan 2018, observation by Aira Francisca Faúndez Fallau (https://www.inaturalist.org/observations/50569276); • Approx. 17 km south of Carrizalillo along route C-503, 28°19'55"S, 69°49'31"W, 10 Jan 2018, observation by Aira Francisca Faúndez Fallau (https://www.inaturalist.org/observations/50569279).

***Cyphocarpusinnocuus*: Chile** • **Coquimbo Region: [Choapa Province**] Reserva Nacional Las Chinchillas, Qda. Los Pilques, 31°28'60"S, 71°04'60"W, 1100 m, 01 Oct 2002, *L. Suarez 1115* (CONC); • [**Elqui Province**] Los Algarrobos, Km. 40–41 Vicuña-Hurtado, 30°16'00"S, 70°39'00"W, 1200 m, 29 Nov 1939, *R. Wagenknecht 18445* (CONC); • Andacolla [Andacollo] Valley, 30°14'30"S, 71°04'16"W, 900 m, Oct 1927, *C. Elliott & W. Balfour Gourlay 98* (K); • Cerro Tololo, 30°10'00"S, 70°47'60"W, 1800 m, 07 Nov 2008, *M. Rosas 6033* (CONC); • Quebrada San Carlos, 30°04'60"S, 70°47'60"W, 700 m, Dec 1974, *Edding & Villagrán s.n.* (CONC); • Quebrada San Carlos, 30°01'60"S, 70°49'00"W, 520 m, 05 Oct 1991, *G. Arancio 91623* (ULS); • 15 Km. Al interior de Guanta, 29°46'60"S, 70°19'00"W, 1850 m, 06 Dec 1991, *G. Arancio 91948* (ULS); Cuesta de Andacollo, 16 Sept 1957, C. *Muñoz 4280* (SGO); • [**Limarí Province**] Los Molles, 30°45'32"S, 70°39'23"W, 1207 m, 15 Nov 1996, *L. Olivares s.n.* (ULS); • Corral Quemado, 30°25'00"S, 71°02'60"W, 1100 m, 30 Oct 1956, *C. Jiles 3092* (CONC); • 6 Km North of Hurtado, 4 Km S of Portezuelo tres cruces, 30°14'17"S, 70°38'47"W, 1800 m, 31 Oct 1997, *T. Ayers et al. 1514* (SGO); • **Valparaiso Region: [Petorca Province**] Cuesta de Alicahue, 32°16'19"S, 70°48'38"W, 1220 m, 06 Nov 2017, *J. Macaya & al. 367* (CONC); Cuesta Alicahue, 32°16'01"S, 70°48'31"W, 1100 m, 09 Nov 1970, *JP. Simon 112* (SGO).

***Cyphocarpuspsammophilus*: Chile** • **Atacama Region: [Huasco Province**] Cruce a observatorio La Silla, 29°14'45"S, 71°00'19"W, 999 m, 08 Oct 1991, *G. Arancio 91604* (ULS); • Cruce a observatorio La Silla, 29°14'39"S, 71°00'02"W, 980 m, 08 Oct 1991, *G. Arancio 91609* (ULS); • Peaje Cachiyuyo al Este, 29°04'60"S, 70°54'00"W, 370 m, 30 Oct 1991, *G. Arancio 91753* (ULS); • Panamericana, Cruce a Observatorio La Silla, 29°04'60"S, 70°54'00"W, 370 m, 23 Sept 1991, *G. Arancio 91658* (CONC); • 1 Km S de Cachiyuyo, 29°02'37"S, 70°54'16"W, 950 m, 22 Oct 1971, *Marticorena & al. 1766* (CONC); • Cachiyuyo, 29°01'60"S, 70°54'00"W, 23 Oct 1965, *F. Behn s.n.* (CONC); • Cachiyuyo, 29°01'60"S, 70°54'00"W, 800 m, 19 Oct 1957, *Ricardi & Marticorena 4460* (CONC); • Camino a los observatorios La Silla y Campana, 29°01'44"S, 70°43'57"W, 1641 m, 23 Sept 1991, *G. Arancio 91650* (ULS); • Agua Amarga, 27.1 Km S of junction to Vallenar, 1.5 Km E of Hwy 5, 28°47'23"S, 70°46'19"W, 1050 m, 02 Nov 1997, *T. Ayers et al. 1553* (SGO); • C-455 Los Morteros, 28°35'24"S, 70°24'25"W, 2062 m, 18 Nov 2022, *C. De Schrevel 577* (CONC); • C-455 Los Morteros, 28°35'24"S, 70°24'25"W, 2062 m, 18 Nov 2022, *C. De Schrevel 578* (CONC); • Huasco. Qda. San Antonio, 28°29'59"S, 70°28'53"W, 1717 m, 14 Dec 2011, *M. Rosas 7869* (ULS).

***Cyphocarpusrigescens*: Chile** • **Atacama Region: [Chañaral Province**] Ruta C-257 al Oeste, 26°40'60"S, 69°43'60"W, 1845 m, 25 May 2007, *L. Letelier & J. Reyes 1360* (ULS); • [**Copiapó Province**] Quebrada Yerbas Buenas, 28°03'19"S, 70°14'48"W, 1200 m, 12 Oct 2010, *M. Rosas 6988* (ULS); • Valle del [Río] Jorquera, 28°00'00"S, 69°54'00"W, 12 Jan 1979, *Gunckel 4056* (CONC); • Quebrada de Totoral (Boquerones), 27°53'60"S, 70°46'60"W, 160–180 m, 24 Nov 1941, *E. Pisano & R. Bravo 793* (CONC, SGO); • Cerro Bandurrias, 27°51'33"S, 70°24'32"W, Dec 1888, *Geisse 9569* (CONC); • Valle del Río Jorquera, 27°49'60"S, 69°45'00"W, 12 Jan 1970, *O. Zöllner 4056* (CONC); • Camino a Lomas Bayas, 27°38'36"S, 70°00'56"W, 1385 m, 18 Oct 2022, *C. de Schrevel 428* (CONC); • Quebrada San Miguel, km 46, 27°30'10"S, 69°45'06"W, 2021 m, 19 Apr 2006, *M. Rosas 3690* (ULS); • Camino Tinogasta, Quebrada Cruz de Cañas, 27°13'00"S, 69°55'00"W, 1100 m, 06 Jan 1973, *Marticorena & al. 473* (CONC); • Hwy 31, 54–56 Km NE of Copiapó, 27°11'25"S, 69°54'28"W, 1165 m, 05 Nov 1997, *T. Ayers et al. 1581* (SGO); • Camino al Salar de Maricunga Km. 56, 27°04'44"S, 69°42'10"W, 1780 m, 31 Jan 1963, *Ricardi & al. 541* (CONC); Quebrada de San Andrés, 27°04'00"S, 69°42'00"W, 1000 m, 03 Nov 1963, *F. Behn s.n.* (CONC); • Quebrada de Puquios, 1885, Geisse (SGO); [**Huasco Province**] Inicio norte Cuesta Pajonales, Km 580, 29°09'49"S, 71°00'03"W, 1015 m, 28 Oct 2002, *M. Muñoz 4250* (SGO); • Cuesta Pajonales, 29°08'60"S, 70°58'00"W, 900 m, 16 Sept 1957, *Ricardi & Marticorena 4382/767* (CONC); • Cuesta Pajonales, 29°07'60"S, 70°58'60"W, 1150 m, 31 Oct 1991, *R. Rodriguez 2763* (CONC); • Cajon del Río Conay, 3 Km al interior de Conay, 28°58'00"S, 70°07'60"W, 1450 m, 13 Oct 1983, *Marticorena 9558* (CONC); • 1.5 Km al E de Conay, 28°55'00"S, 70°55'00"W, 2000 m, 12 Dec 2008, *M. Rosas 6159* (CONC); • Rio Laguna Grande, entre las Papas y Potrero de Toledo, 28°52'60"S, 70°07'60"W, 2000–2400 m, 19 Jan 1983, *Marticorena & al. 83344* (CONC); • Rio Laguna Grande, Entre la Junta de Valeriano y Las Papas, 28°51'00"S, 70°07'00"W, 1800–2000 m, 18 Jan 1983, *Marticorena & al. 83313* (CONC); • R.N.P. Los Huascoaltinos, Qda. Seca, 28°42'59"S, 70°13'00"W, 2067 m, 12 Dec 2008, *Y. Tracol & M. Leon 6278-12* (ULS); • Cuesta Los Morteros, Precordillera, 28°35'33"S, 70°28'57"W, 1940 m, 16 Oct 2011, *Mieres s.n.* (CONC); • Quebrada Baratillo Sur, 28°22'17"S, 71°07'51"W, 55 m, 06 Oct 2017, *C. Delpiano 520* (ULS); • Quebrada Las Máquinas C-461, 28°17'48"S, 70°23'47"W, 954 m, 25 Oct 2022, *C. de Schrevel 464* (CONC); • Sector Higuerita Norte, 28°11'53"S, 70°55'02"W, 418 m, 10 Nov 2008, *A. Stoll & J. Nickerl 1519* (ULS); • Camino a Carrizal Bajo, 28°07'60"S, 71°07'00"W, 250 m, 31 Oct 1991, *G. Arancio 91685* (CONC, ULS); • Camino Canto del Agua, Cerca de Carrizal Bajo, 28°07'17"S, 71°06'11"W, 83 m, 27 Sept 2016, *P. Medina 3142* (CONC); • Camino Totoral a carrizal, 28°04'37"S, 71°03'15"W, 585 m, 28 Oct 1989, *J. Armesto 89734* (ULS); • Cordón Aguadita, Cerro El Volcán, 10 May 1998, *L. Minoletti s.n.* (SGO); • **Coquimbo Region: [Elqui Province**] Rio Seco, Los Choros, Tres Cruces, 30°13'00"S, 70°39'00"W, 700 m, 21 Sept 1975, *O. Zöllner 8333* (CONC); • Cerro Tololo, 30°10'00"S, 70°47'60"W, 1800 m, 26 Nov 1967, *C. Jiles 5126* (CONC); Camino a Hurtado, 30°08'60"S, 70°42'00"W, 1410 m, 23 Oct 2008, *M. Rosas 5895* (ULS); • Camino Río Hurtado-Vicuña, bajando hacia Vicuña, 30°08'32"S, 70°41'27"W, 1460 m, 11 Nov 2017, *J. Macaya & al. 412* (CONC); • 16 Km. N of Portezuelo tres cruces, 30 Km N of Hurtado, 12–14 Km S of Vicuña, 30°08'28"S, 70°41'22"W, 1400 m, 01 Nov 1997, *T. Ayers et al. 1543* (SGO); • 10 Km al sur de Vicuña, en el camino a Hurtado, 30°07'60"S, 70°40'60"W, 800–1000 m, 13 Oct 1940, *G. Looser 4284* (CONC); • Camino Vicuña-Hurtado, Cuesta el Almendro, 30°07'60"S, 70°40'60"W, 1150 m, 10 Oct 1939, *R. Wagenknecht 18452* (CONC); • Sides of Mountains near Payguano [Paihuano], 30°01'47"S, 70°30'48"W, 1841, *Bridges 1298* (E); El Molle, 29°58'60"S, 70°55'60"W, 600 m, Nov 1952, C. Jiles 2347 (CONC); • Quebrada Guanta, 29°49'60"S, 70°24'00"W, 1300 m, 06 Nov 1991, *G. Arancio 91818* (ULS); Cuesta de la viñita, al interior de Marquesa. Camino al Mineral de Arqueros, 29°48'59"S, 70°50'19"W, 20 Sept 1957, *C. Muñoz 4205* (SGO); • Mineral Los Plomos, 16 Km al S de Tres Cruces, 29°31'05"S, 70°56'59"W, 900–1200 m, 03 Nov 1949, *W. Biese 2908* (SGO); • Ruta D-115, 29°27'50"S, 70°42'50"W, 1340 m, 26 Jan 2009, *A. Stoll & G. Arancio 1970* (ULS); • Los Corrales al N, 29°27'45"S, 70°38'29"W, 2028 m, 26 Jan 2009, *A. Stoll & G. Arancio 1983* (ULS); • Cerca Mineral Los Fierros, 29°22'60"S, 71°10'60"W, 540 m, 31 Oct 1961, *R. Wagenknecht 908* (CONC); • 104 Km S of Vallenar, near turnoff to Punta Colorada, 29°21'03"S, 71°02'21"W, 430 m, 01 Dec 1991, *U. Eggli & B.E. Luenberger 1839* (SGO); Rio Turbio, *Volckmann s.n.* (SGO); • [**Limarí Province**] Corral Quemado, 30°25'00"S, 71°02'60"W, 1100 m, 05 Oct 1950, *C. Jiles 3501* (CONC).

### ﻿Key to the species of *Cyphocarpus* (Fig. 6)

**Table:** 

1	Perennial herb; stems compressed so leaves appear to be arranged in a compact rosette; petioles longer than lamina, lamina as long as wide, margins undulate with mucronate teeth (Fig. 6C)	***C.perennis***
–	Annual herbs; stems more or less elongated, never forming a compact rosette; petioles as long or shorter than lamina, lamina at least twice as long as wide, margins not undulate	**2**
2	Glandular indumentum; floral bracts 1–2 mm long, filiform, entire; wings of the upper lobe not elongated (Fig. 6B)	***C.psammophilus***
–	Scabrid to pilose indumentum; floral bracts 20–30 mm long, toothed; wings of the upper lobe notably elongated	**3**
3	Calyx margin serrate (Fig. 6D)	***C.rigescens***
–	Calyx margin entire (Fig. 6A)	***C.innocuus***

**Figure 6.**
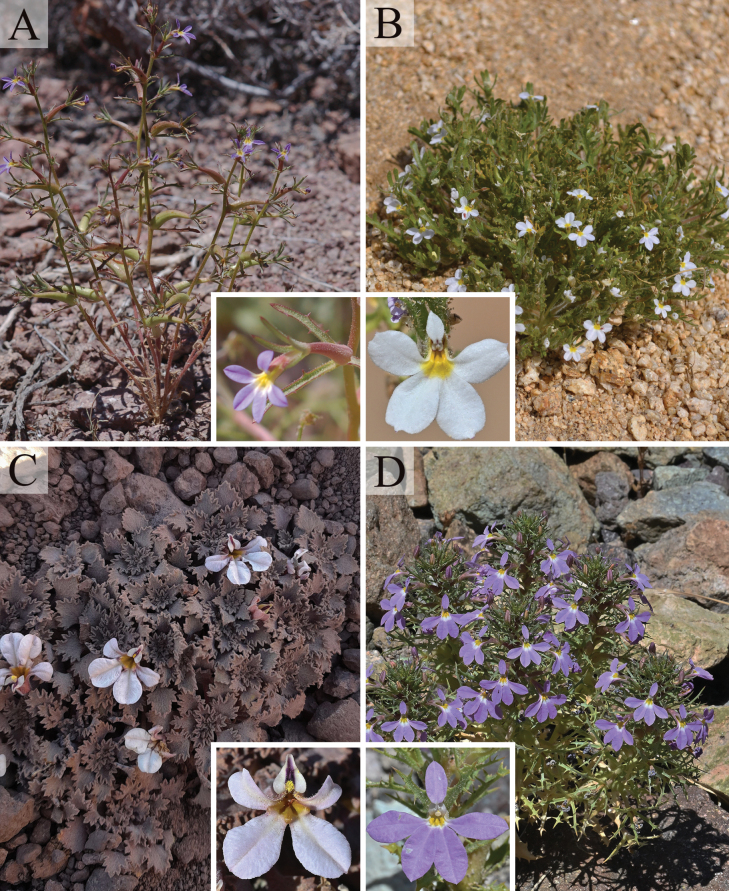
Comparative plate of *Cyphocarpus* species. **A.***C.innocuus* habit and detail of flower (Marcelo Rosas); **B.***C.psammophilus* habit (Marcelo Rosas) and detail of flower (Claire de Schrevel); **C.***C.perennis* habit (Natali Cruz) and detail of flower (Claire de Schrevel); **D.***C.rigescens* habit and detail of flower (Claire de Schrevel).

## ﻿Discussion

Our study confirms the endemism of *Cyphocarpus* to Chile, restricted to the Valparaíso, Coquimbo, and Atacama regions. However, the new species, *Cyphocarpusperennis*, extends the elevational distribution of the genus by about 1,300 meters, reaching up to 3,700 m. This increase in elevation coincides with a shift in life history strategy, as *Cyphocarpusperennis* is perennial unlike the other three annual species in the genus (Lammers 2007). Several plant groups in the Andes present this pattern, such as *Adesmia* (Pérez et al. 2024), *Lupinus* (Hughes and Eastwood 2006), *Leucheria* (Pérez et al. 2020), among others (Hughes and Atchison 2015), where shifts from annual to perennial life forms are correlated with shifts from lowland to montane habitats. The shift to perennial habit is likely an adaptation to the harsh environmental conditions at high elevations, characterized by short growing seasons, frequent frosts, and high climate variability, which reduce the probability of completing the life cycle within a single year (Friedman 2020, Hughes and Atchison 2015, Bliss 1971). Likewise, several comparative studies have shown that perennial life cycles tend to shift to annual life histories in hot and dry conditions (Friedman 2020, Friedman and Rubin 2015).

Our phylogenetic results are consistent with those obtained by Hansen (2016). The topology of both Bayesian and Maximum Likelihood analyses of the nuclear DNA obtained by Hansen (2016) shows Cyphocarpoideae as sister to Nemacladoideae. Within Cyphocarpoideae, our topology is also consistent with both nuclear and plastid topologies obtained by Hansen (2016), showing *Cyphocarpusinnocuus* as sister to *Cyphocarpusrigescens*, and this clade as sister to *Cyphocarpuspsammophilus*. The placement of *Cyphocarpusperennis* within the genus *Cyphocarpus* was initially suggested based on its floral morphology, despite its unusual vegetative morphology. Interestingly, our results position *Cyphocarpusperennis* as sister to all other *Cyphocarpus* species. While our phylogenetic analyses were based solely on nuclear ribosomal DNA sequences, it is acknowledged that the incorporation of plastid or genome-wide data could potentially alter the phylogenetic placement of *Cyphocarpusperennis*. Such data would provide a more comprehensive understanding of its evolutionary relationships and address concerns related to incomplete lineage sorting or hybridization events. Therefore, future studies should consider a multilocus phylogenetic approach to further elucidate the phylogenetic placement and evolutionary history of *Cyphocarpus*.

*Cyphocarpusperennis* is not included in any protected area in Chile. The area where the species thrives is well known for its mining activities, focused mainly in copper and gold. The Atacama Region is one of the most exploited regions of Chile, with approximately 84% of its surface occupied by mining concessions, the highest among Chilean administrative regions (Ministerio de Minería 2022).

The discovery of *Cyphocarpusperennis*, a new endemic of the Andes of the Atacama Desert of Chile, highlights the exceptional biodiversity and high levels of endemicity in this region. It also emphasises the need to increase fieldwork in the area. The Atacama Region is a hotspot of endemism, harbouring nearly 56% of Chile’s endemic vascular plant species, with almost 8% restricted solely to this region (Squeo et al. 2008). Notably, in the past decade, nearly 25% of all newly described Chilean species are known to be found in the Atacama Region, surpassing any other region in the country (https://www.ipni.org/). It is likely that many more undescribed species remain to be discovered within this region. However, several challenges hinder comprehensive biodiversity research in the Atacama region. The steep topography, combined with the unpredictable annual variation in precipitation, severely limits accessibility and complicates fieldwork planning. Furthermore, the lack of accessible roads and the prevalence of privately owned land and restricted access due to mining activities pose substantial logistical and legal obstacles to field research efforts.

## Supplementary Material

XML Treatment for Cyphocarpus perennis

## ﻿Additional information

### Author ORCIDs

Ludovica Santilli https://orcid.org/0000-0001-5429-3441

Philippe Dandois https://orcid.org/0000-0003-1955-1294

Nicolás Lavandero https://orcid.org/0000-0002-2390-5078

Maria Fernanda Perez https://orcid.org/0000-0001-5730-9760

### Data availability

All of the data that support the findings of this study are available in the main text or Supplementary Information.

## Supplementary materials

Supplementary material 1 GenBank accession numbers for the ITS sequences used in this study Data type docx Explanation note GenBank accessions in bold are new to this study. https://binary.pensoft.net/file/1362509This dataset is made available under the Open Database License (http://opendatacommons.org/licenses/odbl/1.0/). The Open Database License (ODbL) is a license agreement intended to allow users to freely share, modify, and use this Dataset while maintaining this same freedom for others, provided that the original source and author(s) are credited.Ludovica Santilli, Natali Cruz, Claire De Schrevel, Philippe Dandois, Nicolás Lavandero, Maria Fernanda Perez
