# Supplementary material for: ﻿Cyphocarpusperennis (Asterales, Campanulaceae, Cyphocarpoideae), a new species endemic to the Andes of the Atacama Desert, Chile
Source: PhytoKeys. 2025 Jun 27;259:111–29. doi: 10.3897/phytokeys.259.155414 (PMC12239027; doi:10.3897/phytokeys.259.155414)
Supplement: Supplementary material 1 — GenBank accession numbers for the ITS sequences used in this study [file phytokeys-259-111_article-155414__-s001.docx]

**Supporting Information**

**Table S1.** GenBank accession numbers for the ITS sequences used in this study. GenBank accessions in bold are new to this study.

| Family | Subfamily | Genus | Species | Author | Voucher | Locality | ITS |
| --- | --- | --- | --- | --- | --- | --- | --- |
| Campanulaceae | Cyphocarpoideae | *Cyphocarpus* | *rigescens* | Miers | Muñoz 4250 (SGO) | Chile: Atacama, Cuesta Pajonales | **PQ059436** |
| Campanulaceae | Cyphocarpoideae | *Cyphocarpus* | *innocuus* | Sandwith | Ayers 1514 (SGO) | Chile: Coquimbo, between Hurtado and Portezuelo Tres Cruces | **PQ059437** |
| Campanulaceae | Cyphocarpoideae | *Cyphocarpus* | *psammophilus* | Ricardi | Ayers 1553 (SGO) | Chile: Atacama, Agua Amarga | **PQ059438** |
| Campanulaceae | Cyphocarpoideae | *Cyphocarpus* | *perennnis* | Santilli & Lavandero | Claire de Schrevel 931 (SGO) | Chile: Atacama, Tierra Amarilla. Cerro Morros Negros | **PV588668** |
| Campanulaceae | Nemacladoideae | *Nemacladus* | *eastwoodiae* | N. Morin & T. Ayers | NRM594 (UC) | - | OK157384 |
| Campanulaceae | Nemacladoideae | *Nemacladus* | *orientalis* | (McVaugh) Morin | LKN10 (ASC) | - | OK157403 |
| Campanulaceae | Nemacladoideae | *Nemacladus* | *pinnatifidus* | Greene | NRM658 (UC) | - | OK157408 |
| Campanulaceae | Nemacladoideae | *Nemacladus* | *ramosissimus* | Nutt. | NRM661 (UC) | - | OK157410 |
| Campanulaceae | Nemacladoideae | *Pseudonemacladus* | *oppositifolius* | (B.L.Rob.) McVaugh | TJA1700 (ASC) | - | OK157426 |
| Campanulaceae | Campanuloideae | *Codonopsis* | *pilosula* | (Franch.) Nannf. | Dong,S.-L. Q557 | - | MH711439 |
